# Moving beyond Aim Three: a need for a transdisciplinary approach to build capacity for economic evaluations in implementation science

**DOI:** 10.1186/s43058-021-00239-1

**Published:** 2021-12-04

**Authors:** Miya L. Barnett, Nicole A. Stadnick, Enola K. Proctor, Alex R. Dopp, Lisa Saldana

**Affiliations:** 1 Department of Counseling, Clinical, & School Psychology, University of California, Santa Barbara, Santa Barbara, CA 93106-9490 USA; 2 Department of Psychiatry, University of California, San Diego, La Jolla, CA 92093 USA; 3 Child and Adolescent Services Research Center, San Diego, CA 92123 USA; 4 UC San Diego Altman Clinical and Translational Research Institute Dissemination and Implementation Science Center, La Jolla, CA 92093 USA; 5 Brown School, Washington University in St. Louis, One Brookings Drive, Campus Box 1196, St. Louis, MO 63130 USA; 6 Department of Behavioral and Policy Sciences, RAND Corporation, 1776 Main Street, Santa Monica, CA 90401 USA; 7 Oregon Social Learning Center, 10 Shelton McMurphey Blvd., Eugene, OR 97401 USA

**Keywords:** Health economics, Economic evaluation, Team science, Capacity building

## Abstract

**Background:**

Understanding the costs and economic benefits of implementation has been identified by policymakers and researchers as critical to increase the uptake and sustainment of evidence-based practices, but this topic remains relatively understudied. Conducting team science with health economists has been proposed as a solution to increase economic evaluation in implementation science; however, these recommendations ignore the differences in goals and perspectives in these two fields. Our recent qualitative research identified that implementation researchers predominantly approach health economists to examine costs, whereas the majority of health economists expressed limited interest in conducting economic evaluations and a desire to be more integrated within implementation science initiatives. These interviews pointed to challenges in establishing fruitful partnerships when health economists are relegated to the “Third Aim” (i.e., lowest-priority research objective) in implementation science projects by their research partners.

**Discussion:**

In this debate paper, we argue that implementation researchers and health economists need to focus on team science research principles to expand capacity to address pressing research questions that cut across the two fields. Specifically, we use the four-phase model of transdisciplinary research to outline the goals and processes needed to build capacity in this area (Hall et al., Transl Behav Med 2:415–30, 2012). The first phase focuses on the *development* of transdisciplinary research teams, including identifying appropriate partners (e.g., considering policy or public health researchers in addition to health economists) and building trust. The *conceptual* phase focuses on strategies to consider when developing joint research questions and methodology across fields. In the *implementation* phase, we outline the effective processes for conducting research projects, such as team learning. Finally, in the *translation* phase, we highlight how a transdisciplinary approach between health economists and implementation researchers can impact real-world practice and policy.

**Summary:**

The importance of investigating the economic impact of evidence-based practice implementation is widely recognized, but efforts have been limited due to the challenges in conducting team science across disciplines. Training in team science can help advance transdisciplinary efforts, which has the potential to increase the rigor and impact of economic evaluations in implementation science while expanding the roles taken by health economists.

Contributions to the literature

- A focus on transdisciplinary research practices can improve collaborations with health economists and implementation scientists.
- Strategies to improve transdisciplinary research may increase the capacity to conduct economic evaluations in implementation science.
- Transdisciplinary research with health economists and implementation science has the potential to move beyond an “Aim 3” approach, which has limited contributions from health economists.
- Strong transdisciplinary research can advance methodological rigor and impact across disciplines.

## Background

"Can you do my Aim 3"?

Economic evaluations remain rare in implementation science even though understanding the costs associated with implementing and sustaining evidence-based practices (EBPs) is necessary for decision-makers [1–3]. Briefly, economic evaluation is a set of methods for comparing the costs and consequences for allocating healthcare resources among alternative services [4], with examples including cost-effectiveness analysis, benefit-cost analysis, and budget impact analysis. Guidance for applying economic evaluation methods to EBP implementation strategies is available, but rarely used [4]. The paucity of economic evaluations in implementation science is influenced by numerous factors including difficulty tracking intervention, implementation, and downstream costs and limited pragmatic costing methods [5, 6]. Further, though multiple researchers have called for increased collaborations between health economists and implementation researchers to improve economic evaluations in implementation science, this suggestion underestimates the misalignment between fields that currently hinders the contributions of health economics within implementation science [7, 8].

In fact, a recent qualitative study our team conducted with implementation researchers and health economists in the USA identified a significant mismatch across fields regarding motivation for conducting economic evaluations [8]: Implementation researchers expressed that they most frequently approach health economists to measure costs associated with implementation strategies, whereas the health economists expressed limited interest in this line of research. As described by one health economist participant, “I don’t want to do cost-benefit analysis. It does seem like implementation science could use someone to come in and do some cost-benefit analysis; I keep hearing it. But I refuse to do it.” These findings reflected a common misunderstanding about the field of health economics, with a perception that researchers were predominately interested in economic evaluations. Although some have an interest in optimizing efficiency and costs and using this information to advance the scale-up of existing interventions, many health economists predominately focus on empirical models of behavior. Therefore, our study pointed to the value of expanding collaborations with health economists in implementation science beyond economic evaluations. Further, our qualitative results revealed that even when health economists did partner with implementation researchers to conduct economic evaluations, they described the resources (e.g., Co-I effort, research personnel) needed to complete these evaluations were often underestimated. On federally funded research grants, economic evaluations often were “Aim 3,” which can receive fewer resources and lower priority and often is the aim that is cut by the principal investigators throughout the course of the grant period. In combination, these findings suggested areas for improvement in conducting transdisciplinary research across implementation science and health economics.

Even though implementation science is by definition a transdisciplinary field, we argue that the predominant “Aim 3” approach to conducting economic evaluations has led to a limited capacity to answer research questions related to implementation costs. Further, implementation science has not fully integrated the expertise of health economists, which extends beyond economic evaluations and could further advance the methods and research questions considered in implementation research. Cross-disciplinary collaborations have different levels of integration, ranging from being multidisciplinary to transdisciplinary [9, 10]. In a multidisciplinary team, researchers from different disciplines work within their respective fields and then apply their findings to a common research goal. This is frequently the “Aim 3” approach, where health economists conduct the economic evaluation separately from the primary aims of the grant, which focus on the effectiveness of the implementation strategy and/or EBP. On the other end of the spectrum, transdisciplinary research combines expertise and methodologies from multiple disciplines into a hybrid discipline with new conceptual frameworks, methodologies, models, and theories that can accelerate innovation and impact on complex social problems [10, 11]. That is to say that while researchers enter into transdisciplinary research with their own discipline’s perspective, they emerge from this work with new shared perspectives (e.g., developing a new method to conduct an economic evaluation within implementation studies). For example, researchers and educators have adopted the transdisciplinary approach of Human Systems Engineering, which integrates engineering with psychology, to increase the capacity of engineers to address human factors that impact real-world technological problems (e.g., cybersecurity) [12].

In this paper, we contend that a focus on conducting successful transdisciplinary research is critical to build capacity for economic evaluations within implementation science and extend the contributions from health economists on the methodology in the field. Though the benefits for transdisciplinary research are numerous (e.g., producing more impactful and innovative research), the process of forming teams and working together can be challenging, which has prompted research on the processes and strategies that are needed for team science to function successfully [9, 11, 13, 14]. Team science is defined as a collaboration among multiple scientists from diverse disciplines who conduct interdependent tasks to accomplish a goal [9]. We use Hall et al.’s four-phase model of transdisciplinary research to propose how implementation study teams can fully integrate health economists, with considerations for how capacity challenges regarding economic evaluations can be addressed. This model was selected because it provides guidance on scientific goals and key processes across various phases of transdisciplinary research focused on addressing social and public health challenges.

## The four-phase model of transdisciplinary research

Drawing on the research of how to effectively conduct team science, the four-phase model includes key goals and team processes across the phases of (1) *development*, (2) *conceptualization*, (3) *implementation*, and (4) *translation* [15]. Not unlike implementation process frameworks [16], the four-phase model for developing a team science approach recognizes that progression through the phases is not linear, and revisiting phases may be appropriate and needed. For example, if new research questions are identified in later stages, different expertise might be needed that could require the addition of new team members (i.e., a return to the development phase). In the following sections, we apply recommendations from this model to the fields of implementation science and health economics and propose opportunities to enhance integration across fields given calls for greater collaborations across these fields to improve measurement of implementation costs [17–19]. Table 1 summarizes the recommendations regarding the goals, team members, and processes in each of the phases of transdisciplinary implementation science with health economists.

**Table 1:** Four phases of transdisciplinary implementation research with health economists

	Development	Conceptualization	Implementation	Translation
**Goals**	Establish a shared understanding of the scientific or societal problem and mission of the group	Develop research questions, hypotheses, and study designs that integrate and extend approaches from implementation science and health economics	Launch, conduct, and refine the planned transdisciplinary research project	Research findings inform policy and practice related to the implementation and sustainment of real-world programs.
**Team members**	• Health economists • Health services researchers • Implementation researchers • Community stakeholders • Clinicians • Intervention researchers	• Health economists • Health services researchers • Implementation researchers • Advocacy groups • Organizational leadership • Policymakers • Clinicians • Intervention researchers	• Health economists • Health services researchers • Implementation researchers • Community stakeholders • Master’s level researchers (e.g., from public health, public policy, economics) • Trainees • Billing department	• Health economists • Health services researchers • Implementation researchers • Community stakeholders • Organizational leadership • Policymakers • Financing groups (e.g., insurers)
**Processes**	• Develop shared mission and goals • Reflect on methodological approaches in each field • Identify and discuss the “coins of the realm” for all members of the team • Promote psychological safety and express appreciation for the expertise of different disciplines	• Develop a shared language on terminology related to methods and outcomes • Identify and understand the expertise of different members of the group	• Conduct regular team meetings to support integration and team learning • Manage conflict by allowing for respectful debate across discipline	• Identify how economic evaluations can inform the financing of implementation efforts. • Adapt the team as needed.

*Note*: Adapted from Hall et al. (2012)

### Development phase

The primary goal of the development phase is to establish a shared understanding of the scientific or societal problem, including what concepts fall inside and outside the problem’s boundaries, and ultimately determine the mission of the research group. This step is particularly critical for successful transdisciplinary research between implementation researchers and health economists given the disciplinary differences in conceptualizing research questions and methods. When identifying health economists who might be interested in transdisciplinary implementation research, it is important to recognize that the field of economics is made up of multiple subspecialties (e.g., welfare economics, game theory), so it is important to identify health economists with specialization and interests relevant to implementation science—such as in economic evaluations. Additionally, it has been proposed that behavioral economics is a strong fit for designing implementation strategies, highlighting the role economists can have within implementation science beyond conducting economic evaluations [20]. Furthermore, our qualitative findings suggest that identifying a shared interest in a societal problem (e.g., substance abuse interventions) and the potential to conduct research that impacts policy may be a point of convergence between health economists and implementation researchers [8].

An important question to address for transdisciplinary teams in implementation science and health economics is how do researchers across these disciplines have the opportunity to meet and collaborate? A key opportunity could exist for researchers located at institutions with Centers for Translational Science Award (CTSA) Programs, which are funded by the National Institute of Health to “speed the translation of research to improved patient care [21].” Currently, there are more than 50 CTSA sites across the USA, which are typically in schools of medicine with members spanning many disciplines. CTSAs can play a critical role in supporting the dissemination and implementation science training and research broadly and can be an excellent way for researchers across disciplines to come together [22]. Recognizing the need for team science to advance translational research, the UC San Diego Altman Clinical and Translational Research Institute recently added a Team Science Core to build capacity for clinical and implementation researchers to learn about and integrate team science principles, assemble teams, and evaluate team functioning. Similarly, cross-university centers and institutes bring researchers together across fields. Examples include NIH-funded initiatives such as the National Institute of Mental Health Advanced Laboratories for Accelerating the Reach and Impact of Treatments for Youth and Adults with Mental Illness (ALACRITY) program or the National Institute of Allergy and Infectious Diseases/NIH Implementation Science, Coordination, Consultation, & Collaboration Initiative (ISC^3^I) [20, 21]. Training programs that have focused on increasing capacity in dissemination and implementation science, such as the Implementation Research Institute, also offer a rich opportunity to foster collaborations across disciplines [23–25]. However, additional efforts might be required to increase the representation of health economists within these spaces to foster these collaborations. For example, in developing a small conference focused on reducing mental health disparities, efforts were made to invite international health economists and implementation researchers, which led to rich opportunities to share disciplinary perspectives [26].

Identifying a shared mission and goals is a critical process within the development phase to increase the motivation of team members. Though research teams frequently are formed with a project in mind (e.g., when preparing a grant proposal), spending time in this phase could enhance integration between the fields of health economics and implementation science. This is especially important given the findings that health economists may have less motivation to conduct economic evaluations within implementation studies than implementation researchers have in these evaluations being completed [8]. Further, it can be valuable to develop *critical awareness*, in which all research collaborators reflect on the methodological strengths and weaknesses of their respective fields. For example, health economists may focus on identifying costs that are nationally representative, whereas implementation researchers may be more focused on the local context [3].

Similar to processes that occur between researchers and community stakeholders in collaborative research, a cultural exchange occurs in which different groups come into a project with different knowledge, attitudes (e.g., motivations), and practices that result in an overarching model that transcends disciplines. The goal is aims that are shared by all parties, with equal motivation and vision for what will advance the study questions. In order for these cultural exchanges to be successful, different group members need to have perceived reciprocity, and everyone should get something desirable from the interaction [27]. That is to say, the benefits from the transdisciplinary collaboration have to be explicit and relevant to each discipline, with rewards that are commensurate with the required effort. When disciplines have different “coins of the realm,” this work is challenging. These disciplinary differences were noted in interviews with health economists, in that there has traditionally been less emphasis on collaborative research in economics, there is a greater emphasis on publishing in economic journals for career advancement, and having salary covered by grants is not always needed [8]. As one health economist described, “If you’re interested in implementation science you’re never going to get into the Quarterly Journal of Economics, it’s just not gonna happen. And they [economics departments] don’t even really value a JAMA piece.”

Process-wise, an essential foundational element of this phase is promoting a strong sense of *psychological safety* among research team members. Psychological safety is an organizational characteristic in which individuals feel comfortable sharing their opinions and experiences without fear of judgment or retribution [28]. This includes making sure that team members feel like their discipline is appreciated and spending time asking questions and expressing ideas to gain a better understanding of disciplinary cultures, approaches, and models. Notably, health economists have expressed concerns that their methodological expertise is not understood or utilized, which could lead to feeling underappreciated [8]. Therefore, spending dedicated time to understand the unique and complementary contributions across fields is critical for improved transdisciplinary research.

### Conceptualization phase

The primary goal of the conceptualization phase is to develop research questions, hypotheses, and study designs that integrate disciplinary perspectives. According to Hall et al., for work to truly become transdisciplinary, it is important for collaborators to “let go of discipline-based lines of inquiry and embrace the goal of integration” (p. 420). Notably, the conceptualization phase points to the importance of implementation science teams integrating the perspectives of multiple disciplines, including health economists, into the formation of research questions and designs that reflect the integrative nature of the project. A part of these planning discussions is determining if the time frame of the study is appropriate to the questions asked, since long-term outcomes might be needed for some economic evaluations. Beyond health economists, other fields (e.g., accounting) and stakeholders focused on financing (e.g., healthcare payors) bring important perspectives that are needed to truly expand the impact and extent of economic evaluations within implementation science and should also be included. This runs counter to the “Aim 3 approach” of identifying a collaborator to conduct an economic evaluation of a fully formed research idea and is consistent with past recommendations to start research partnerships early in the idea-generating period of the project and thus have shared understandings from which to collaborate on the methodological approach used in a study [8, 19, 29].

In the conceptual phase, it is critical that researchers develop a shared language, so that all team members are equally able to understand the research approach and conceptual models being used. For economic evaluations in implementation science, this is critical as the point of evaluation or interest around costs can vary across disciplines. For example, implementation researchers and practitioners may find more value in understanding costs to deliver or sustain an EBP or implementation strategy, whereas health economists might be more likely to focus on a broad, societal cost perspective or on clinical outcomes, such as quality-adjusted life-years (QALYs). There is a concrete opportunity to offer a terminology primer for both implementation researchers and health economists to facilitate shared language, such as that provided in this Special Collection in the Glossary.

Though the developmental and conceptual phases aim to enhance integration, there is still a recognition that members of the team will have different areas of expertise. Therefore, the development of *compilational transactive memory*—building an understanding of who on the team has what expertise and relying on that expertise when making relevant key decisions for the research—is another key process in this phase [15]. Not only will gaining shared understanding of the roles and expertise improve efficiency on the project, but it might help to build opportunities for future collaborations across different team members.

### Implementation phase

The implementation phase of Hall et al.’s model of transdisciplinary research is focused on conducting the research project, including processes that facilitate more formal involvement in the project based on specified roles. It is important to jointly establish appropriate frequency and formats for communication, roles, and procedures. Weekly and daily work schedules may differ significantly across fields. For example, teams with clinician researchers may need to shift meeting times to accommodate clinical schedules. Collaborators should strive for balance and reciprocal effort investment within these meetings.

An important skillset for leaders of transdisciplinary teams includes *conflict management*. Though all teams can experience conflict, this is especially important when working across disciplinary cultures, beliefs, and methods. Beyond divergent frameworks and methodologies, communication styles can vary across disciplinary cultures and individuals. For example, the time spent on rapport building, efficiency, and tolerance for criticism may differ across team members. Managing conflict is not the same as avoiding it, and indeed space for debate can help advance the integration across disciplines. Transdisciplinary teams require strong leadership from individuals skilled in fostering cooperation and free exchange of ideas, in building consensus and discouraging competition and defensiveness among team members [30]. Processes need to be established that provide time and opportunity for team members to express ideas, similar to those required for team processes to adapt interventions for implementation. For example, Hasche and colleagues established a virtual meeting process, led by the principal investigator, ensuring that each member of the transdisciplinary team could express their unique and often divergent views. During the meetings, detailed notes were recorded to capture each decision point along with the rationale for the decision reached. Following meetings, detailed notes were sent to team members. At the start of the next conference call, decisions at prior meetings were revisited, and opinions about the decisions were invited. This process was repeated until consensus was achieved [31].

*Team learning* refers to the acquisition of knowledge, skills, and performance capabilities of individuals within a team [32]. At times, there can be a pull to have few contacts between team members to maximize efficiency, and projects that started with a transdisciplinary orientation and integrative research questions unintentionally are executed in discipline-specific segments by separate investigators. This is especially common if a collaborator on the team has been given a limited role and effort on a grant, as can occur for an investigator responsible for “Aim 3.” Without opportunities for systematic reflection and refinement of team performance, goals, processes, and knowledge exchange, team learning and innovation can be limited. A concrete example would be to routinely invite health economic collaborators to regular research team meetings, especially during the early implementation of a project when methods are being refined.

During this implementation phase, in order to address limitations that can occur with budgets and schedules, it is important to determine who is conducting different tasks related to the economic evaluation. When conducting economic evaluations, it is important to staff the team with individuals who are interested, capable, and well-trained in the collection of the cost data. Team members who are supporting cost data collection and analyses, such as Master’s-level researchers or trainees (e.g., post-doctoral fellows) without a health economics background, may require a certain level of supervision by a health economist. Therefore, when planning collaborative research projects, it is important to allocate appropriate resources (i.e., effort and staffing) so that health economists can contribute to the transdisciplinary processes that allow for team learning, along with the study execution. These resources will be dependent on the complexity of the cost data that is collected, as some approaches are more pragmatic or efficient, which can reduce the burden of conducting economic evaluations in implementation research [6]. Other team members (e.g., clinical or implementation experts) should also have defined roles in completing the economic evaluation and interpreting its findings.

### Translational phase

The translational phase is in many ways of primary interest to both implementation researchers and health economists—it focuses on how study findings can have a real-world impact on clinical practice, healthcare systems, and policy. It is important for transdisciplinary research teams to plan for the translation of the findings of economic evaluation outcomes to relevant audiences. As with many implementation studies, this highlights the need to include perspectives from multiple stakeholders, including organizational leaders, policymakers (e.g., government administrators), and financing groups (e.g., insurers). This may be especially imperative in settings with limited resources, as underestimating costs in these contexts is even more likely to lead to failed implementation [19].

The translational phase can highlight new opportunities for cross-disciplinary and cross-sector collaborations. An EBP or implementation strategy can reduce the overall costs to a system of care, but still not be feasible if there is no way to finance it. For example, task-sharing with community health workers has been identified as a strategy that can increase the quality of care, while decreasing healthcare costs. However, services provided by community health workers are rarely reimbursable, which severely limits opportunities for scale-up and sustainment [33]. Therefore, once implementation costs are identified, financing strategies are needed to provide funding that covers those costs and additional costs that are needed to expand capacity in the EBP, which may require additional partnerships with policymakers and insurers for economic evaluations to impact practice [34]. Another paper in this special collection by Dopp and colleagues provides several case examples of how economic evaluations can be translated into implementation financing strategies, and a team science approach to economic evaluation could certainly accelerate such translation of findings [35]. Furthermore, healthcare is resplendent with examples of low-value practices that need to be de-implemented. However, if a healthcare organization’s budget depends on providing that practice, incentives for de-implementation decrease [36]. In the case of ineffective interventions or implementation strategies, a full economic evaluation (e.g., cost-effectiveness analyses) might not be appropriate, but cost data could still inform how to tailor implementation to improve care in the future [37]. Moreover, understanding the costs associated with ineffective strategies can be important information when developing strategies for de-implementation. These examples highlight that multiple economic factors will impact decision-makers in regard to EBP implementation, which extend beyond the cost of implementation or the cost-effectiveness of an EBP or implementation strategy, pointing to additional foci for translational work that health economists can help advance within implementation science.

## Conclusions

Overall, these recommendations highlight opportunities to move from predominately using a multidisciplinary approach in collaborations between implementation science and health economics, to becoming truly transdisciplinary. By attending to the goals and processes in each phase of both health economists and implementation scientists, there is the potential to enhance methodological rigor in implementation research, not only through economic evaluations, but with additional areas of expertise from health economists related to understanding stakeholder and organizational behavior. Implementation researchers are well-positioned to lead these transdisciplinary efforts, given their understanding and emphasis on team science and cultural exchange with community stakeholders [13, 27]. Implementation science is inherently transdisciplinary, with no consistent university or field claiming to be its home [24]. Yet, even within implementation science, it has been identified that researchers from the same discipline (e.g., psychology) frequently collaborate together [38], with secondary questions being pursued through ancillary collaborations. This limited approach suggests a need to foster more integration across disciplines, including health economics, with a goal of achieving shared visions for advancing study questions. Accordingly, training programs in dissemination and implementation need to provide examples and guidance for transdisciplinary work. Training programs should include sessions or panels displaying transdisciplinary partnerships in action, demonstrate the value-added of transdisciplinarity, and identify and coach trainees in the skills for such partnerships. We envision building an approach to collaborations across health economists and implementation researchers that maximizes the intellectual contributions and real-world impact of all team members. An additional benefit for health economists could be to finally escape the “Aim 3” designation on implementation science projects, both broadening and deepening the potential contributions that these valuable colleagues make to implementation science.

## Data Availability

The interviews generated and analyzed during the current study are not publicly available to protect the identities of participants, who are members of the academic community, but are available from the corresponding author on reasonable request.

## References

[1] Pegg SL, Walsh LM, Becker-Haimes EM, Ramirez V, Jensen-Doss A (2019). Money makes the world go ‘round: a qualitative examination of the role funding plays in large-scale implementation and sustainment of youth evidence-based practice. Psychol Serv..

[2] Saldana L, Chamberlain P, Bradford WD, Campbell M, Landsverk J (2014). The cost of implementing new strategies (COINS): a method for mapping implementation resources using the stages of implementation completion. Child Youth Serv Rev.

[3] Wagner TH (2020). Rethinking how we measure costs in implementation research. J Gen Intern Med.

[4] Eisman AB, Kilbourne AM, Dopp AR, Saldana L, Eisenberg D (2019). Economic evaluation in implementation science: making the business case for implementation strategies. Psychiatry Res..

[5] Dopp AR, Mundey P, Beasley LO, Silovsky JF, Eisenberg D (2019). Mixed-method approaches to strengthen economic evaluations in implementation research. Implement Sci.

[6] Cidav Z, Mandell D, Pyne J, Beidas R, Curran G, Marcus S (2020). A pragmatic method for costing implementation strategies using time-driven activity-based costing. Implement Sci.

[7] Smith MW, Barnett PG (2008). The role of economics in the QUERI program: QUERI Series. Implement Sci.

[8] Barnett ML, Dopp AR, Klein C, Ettner SL, Powell BJ, Saldana L (2020). Collaborating with health economists to advance implementation science: a qualitative study. Implementation Science Communications.

[9] Stokols D, Hall KL, Taylor BK, Moser RP. The science of team science. Overview of the field and introduction to the supplement. Am J Prev Med. 2008;35;S77–S89. 10.1016/j.amepre.2008.05.002 18619407

[10] Rosenfield PL (1992). The potential of transdisciplinary research for sustaining and extending linkages between the health and social sciences. Soc Sci Med.

[11] Falk-Krzesinski HJ, Börner K, Contractor N, Fiore SM, Hall KL, Keyton J (2010). Advancing the science of team science. Clin Transl Sci.

[12] Roscoe RD, Becker DV, Branaghan RJ, Chiou EK, Gray R, Craig SD (2019). Bridging psychology and engineering to make technology work for people. Am Psychol.

[13] Aarons GA, Reeder K, Miller CJ, Stadnick NA (2019). Identifying strategies to promote team science in dissemination and implementation research. J Clin Transl Sci..

[14] Hall KL, Vogel AL, Serrano KJ, Fiore SM (2018). The science of team science: a review of the empirical evidence and research gaps on collaboration in science human adaptations in extreme environments view project. Am Psychol.

[15] Hall KL, Vogel AL, Stipelman BA, Stokols D, Morgan G, Gehlert S (2012). A four-phase model of transdisciplinary team-based research: goals, team processes, and strategies. Transl Behav Med.

[16] Moullin JC, Dickson KS, Stadnick NA, Rabin B, Aarons GA (2019). Systematic review of the Exploration, Preparation, Implementation, Sustainment (EPIS) framework. Implement Sci.

[17] Roberts SLE, Healey A, Sevdalis N (2019). Use of health economic evaluation in the implementation and improvement science fields - a systematic literature review. Implement Sci..

[18] Knies S, Severens JL, Brouwer WBF (2019). Integrating clinical and economic evidence in clinical guidelines: more needed than ever!. J Eval Clin Pract.

[19] Sohn H, Tucker A, Ferguson O, Gomes I, Dowdy D (2020). Costing the implementation of public health interventions in resource-limited settings: a conceptual framework. Implement Sci..

[20] Beidas RS, Buttenheim AM, Mandell DS (2021). Transforming mental health care delivery through implementation science and behavioral economics. JAMA Psychiatry..

[21] CTSA Program Hubs | National Center for Advancing Translational Sciences [Internet].

[22] Dolor RJ, Dolor RJ, Proctor E, Stevens KR, Boone LR, Meissner P (2020). Dissemination and implementation science activities across the Clinical Translational Science Award (CTSA) Consortium: report from a survey of CTSA leaders. J Clin Transl Sci..

[23] Proctor EK, Chambers DA (2017). Training in dissemination and implementation research: a field-wide perspective. Transl Behav Med.

[24] Proctor EK, Landsverk J, Baumann AA, Mittman BS, Aarons GA, Brownson RC (2013). The implementation research institute: training mental health implementation researchers in the United States. Implement Sci.

[25] Padek M, Mir N, Jacob RR, Chambers DA, Dobbins M, Emmons KM (2018). Training scholars in dissemination and implementation research for cancer prevention and control: a mentored approach. Implement Sci.

[26] Stadnick NA, Aarons GA, Blake L, Brookman-Frazee LI, Dourgnon P, Engell T, et al. Leveraging implementation science to reduce inequities in children’s mental health care: highlights from a multidisciplinary international colloquium. BMC Proc. 2020;14:1–12. Springer Science and Business Media LLC. 10.1186/s12919-020-00184-2 PMC7132860 32280371

[27] Palinkas LA, Aarons GA, Chorpita BF, Hoagwood K, Rady JL, Hosptial C (2009). Cultural exchange and the implementation of evidence-based practices: two case studies. Res Soc Work Pract.

[28] Edmondson AC, Lei Z (2014). Psychological safety: the history, renaissance, and future of an interpersonal construct. Annu Rev Organ Psych Organ Behav.

[29] Murphy SM, Leff JA, Linas BP, Morgan JR, McCollister K, Schackman BR (2018). Implementation of a nationwide health economic consultation service to assist substance use researchers: lessons learned. Subst Abus..

[30] Gehlert S, Browne T, Haire-Joshu D, McBride TD (2013). Transdisciplinary training and education. Transdisciplinary public health: research, education, and practice.

[31] Hasche LK, Lenze S, Brown T, Lawrence L, Nickel M, Morrow-Howell N (2014). Adapting collaborative depression care for public community long-term care: using research–practice partnerships. Adm Policy Ment Health Ment Health Serv Res.

[32] Kozlowski SWJ, Ilgen DR (2006). Enhancing the effectiveness of work groups and teams. Psychol Sci Public Interest.

[33] Cross-Barnet C, Ruiz S, Skillman M, Dhopeshwarkar R, Singer R, Carpenter R (2018). Higher quality at lower cost: community health worker interventions in the health care innovation awards. J Health Disparities Res Pract..

[34] Dopp AR, Narcisse M-R, Mundey P, Silovsky JF, Smith AB, Mandell D (2020). A scoping review of strategies for financing the implementation of evidence-based practices in behavioral health systems: state of the literature and future directions. Implement Res Pract.

[35] Dopp AR, Kerns SEU, Panattoni L, Ringel JS, Eisenberg D, Powell BJ (2021). Translating economic evaluations into financing strategies for implementing evidence-based practices. Implement Sci.

[36] Prusaczyk B, Swindle T, Curran G (2020). Defining and conceptualizing outcomes for de-implementation: key distinctions from implementation outcomes. Implement Sci Commun..

[37] Weinstein MC, Skinner JA (2010). Comparative effectiveness and health care spending—implications for reform. N Engl J Med.

[38] Norton WE, Lungeanu A, Chambers DA, Contractor N (2017). Mapping the growing discipline of dissemination and implementation science in health. Scientometrics.

